# A case of anastomotic recurrence 12 years after intersphincteric resection for anorectal malignant melanoma

**DOI:** 10.1007/s12328-025-02140-z

**Published:** 2025-05-04

**Authors:** Sato Nishida, Tatsuya Shonaka, Tomohiro Takeda, Masahide Otani, Mizuho Ohara, Chikayoshi Tani, Manami Hayashi, Tomoe Nakagawa, Kimiharu Hasegawa, Hideki Yokoo

**Affiliations:** 1 Division of Gastrointestinal Surgery, Department of Surgery, Asahikawa Medical University, 2-1 Midorigaoka Higashi, Asahikawa, Hokkaido 078-8510 Japan; 2 Division of Hepatobiliary Pancreatic and Transplantation Surgery, Department of Surgery, Asahikawa Medical University, Asahikawa, Japan; 3 Department of Diagnostic Pathology, Asahikawa Medical University Hospital, Asahikawa, Japan; 4 Department of Dermatology, Asahikawa Medical University, Asahikawa, Japan

**Keywords:** Anorectal malignant melanoma, Local recurrence, Intersphincteric resection, Abdominoperineal resection, *BRAF*

## Abstract

Anorectal malignant melanoma (AMM) is a rare disease with a poor prognosis, accounting for < 1.0% of all malignant melanomas and a 5-year survival rate of 19.2%. The treatment is mainly surgical, and lymph-node dissection is often performed. Cases of recurrence after a prolonged period (> 10 years), as in the present case, are rare. The patient was an 80-year-old woman who underwent laparoscopic intersphincteric resection with bilateral lateral lymph-node dissection for the diagnosis of primary AMM of the lower rectum at X – 12 years. The pathology was pStage III and the resection margins were negative. Twelve years after the initial surgery, in year X, the patient visited our hospital with the chief complaint of discomfort due to a tumor in the anorectal region. Biopsy revealed a recurrence, and laparoscopic abdominoperineal resection was performed. Based on the pathological findings, the patient was diagnosed with local recurrence of the anastomotic anal canal. Both the first and second specimens were negative for *BRAF* V600E/K mutation. Four months have passed since the surgery, and the patient continued to receive nivolumab without recurrence. Long-term local follow-up is necessary when the anal canal is preserved during AMM surgery.

## Introduction

Anorectal malignant melanoma (AMM) is a rare disease with a poor prognosis, accounting for < 1% of all malignant melanomas and with a 5-year survival rate of 19.2% [1]. The treatment is mainly surgical and often involves lymph-node dissection [2]. The main surgical resection options are abdominoperineal resection (APR) and local excision [1]. APR completely removes the distal colon, rectum, and anal sphincter, thus resulting in a permanent colostomy [3]. Intersphincteric resection (ISR), which is rarely performed, was performed in this case. ISR is characterized by partial, subtotal, and total resection of the internal anal sphincter via intersphincteric dissection. Coloanal anastomosis is always hand-sewn [4]. ISR preserves the external anal sphincter and defecation function [3, 4]. Relative to APR in the treatment of rectal cancer, the ISR group has equal 5-year disease-free survival and 5-year local recurrence-free survival [5]. AMM is known to have a poor prognosis owing to early hematogenous and lymphogenous metastases [6], with a median recurrence period of 9 months after surgical resection, with most cases recurring within a year [7]. Cases of recurrence of malignant melanoma after > 10 years are rare, as in our patient. The recurrence rates vary depending on the surgical technique, with local excision being riskier than abdominoperineal resection [1]. Owing to its rarity, prospective randomized trials are difficult to produce and have not yet demonstrated an ideal therapy or follow-up protocols. Genetically, 40–50% of all melanoma patients harbor an activating *BRAF* mutation, mostly *BRAF* V600E, but there is little information on AMM [8]. We herein report a case of anastomotic recurrence 12 years after AMM surgery and summarize the characteristics and treatment of AMM.

## Case report

**Fig. 1:**
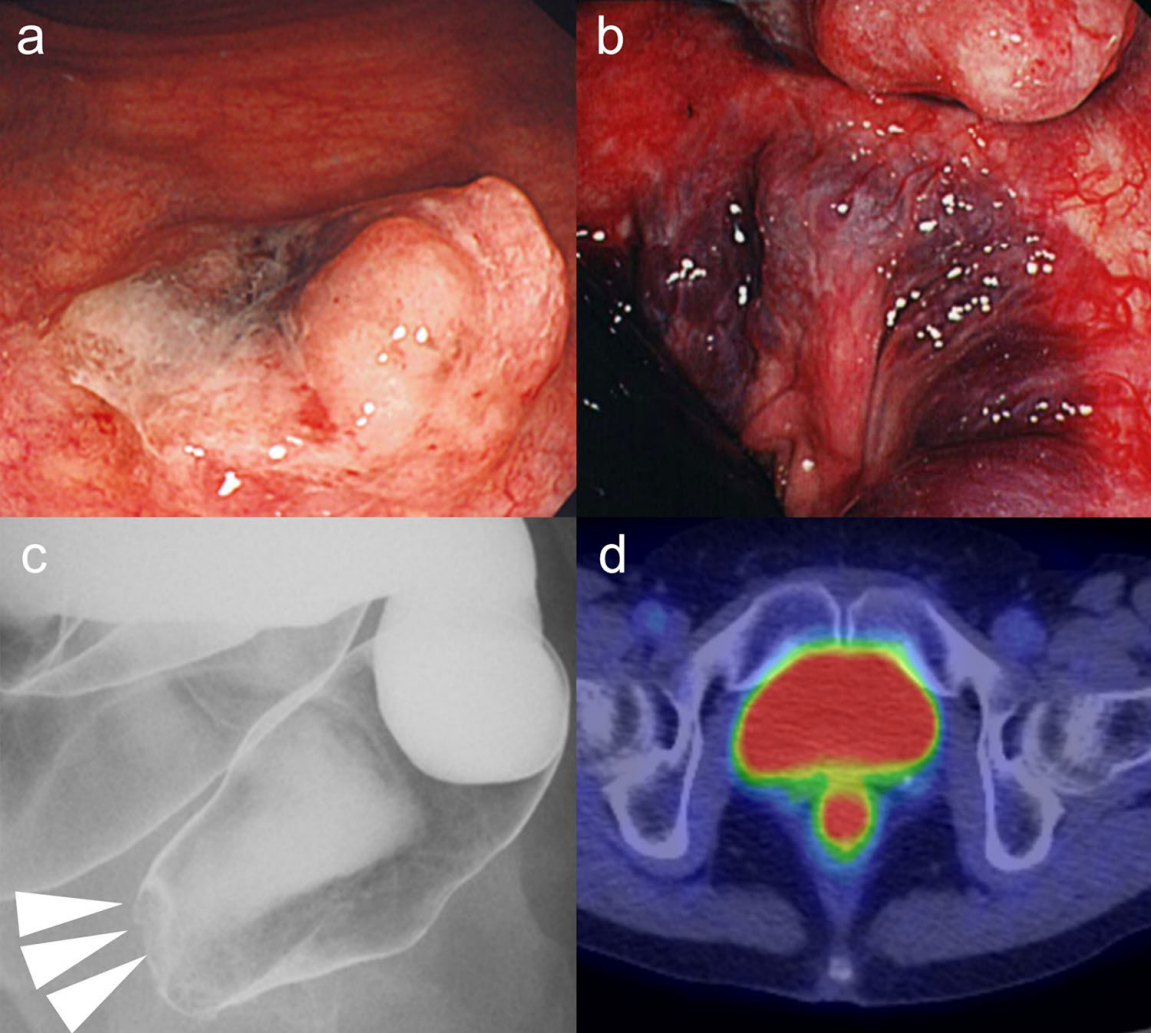
Results of the examination before the first surgical treatment. **a** Colonoscopy revealed a 20 × 13 mm lesion in the lower rectum (4 cm from the anal verge) with a blackish depression in the center. **b** Colonoscopy, revealed a lesion directly above the dentate line. **c** Gastrointestinal enema showing a contrast defect in the anterior wall of the lower rectum. **d** PET showed a localized abnormal accumulation of Max SUV: 6.4–8.6 in the lower rectum

An 80-year-old woman visited our hospital 12 years previously with a chief complaint of bleeding during defecation. At that time, lower gastrointestinal endoscopy detected a 20 × 13 mm lesion in the lower rectum, located 4 cm from the anal verge, which exhibited a blackish central depression (Fig. 1a, b). A gastrografin enema revealed a contrast defect in the anterior wall of the lower rectum (Fig. 1c). The patient was subsequently referred to our department because of a suspected malignant lymphoma. A computed tomography (CT) scan showed a hyperabsorptive area in the rectum in the 12 o’clock direction, and a positron emission tomography (PET) scan identified localized abnormal accumulation with a maximum standardized uptake value (SUV) of 6.4–8.6 in the same area (Fig. 1d). MRI revealed an area of high signal intensity in the same region on DWI, with no evidence of invasion of the surrounding adipose tissue or lymph-node enlargement. A histopathological examination revealed a substantial proliferation of atypical cells with multivesicular reticulum and large nuclei, while immunostaining was positive for S-100, CD57, and Melan A. Based on these findings, the patient was diagnosed with AMM.

**Fig. 2:**
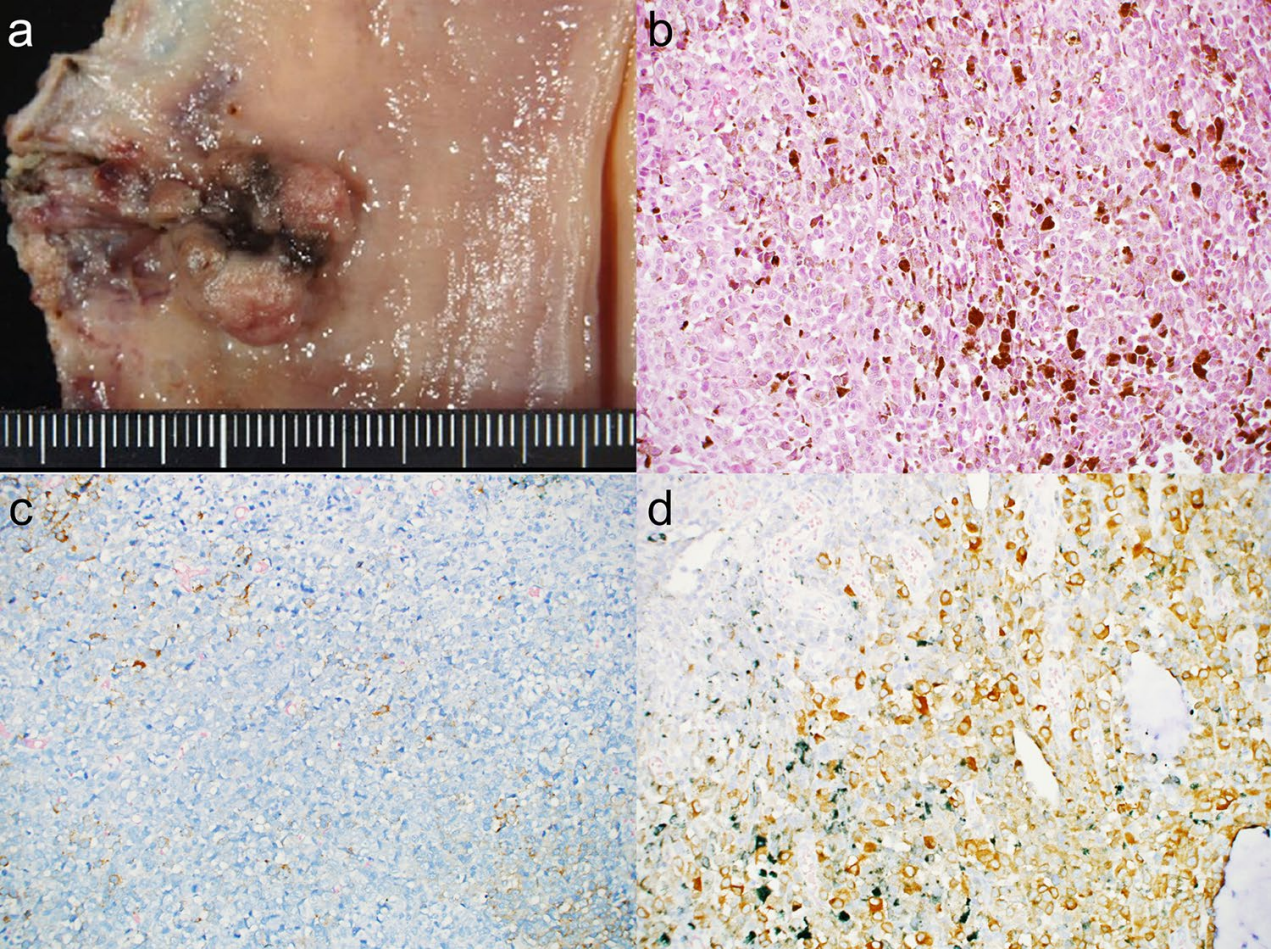
First surgical specimen and pathohistological findings. **a** Surgical specimen, dark-colored, raised lesion measuring 20 × 13 mm directly above the dentate line. **b** Hematoxylin and eosin staining showing a substantial proliferation of atypical cells with multivesicular reticulum and large nuclei (magnification, 20 ×). **c** Immunohistochemical staining of tumor cells for HMB-45 was positive (magnification, 20 ×). **d** Immunohistochemical staining of Melan A tumor cells was positive (magnification, 20 ×)

Although APR was initially recommended, the patient declined to undergo colostomy. Therefore, ISR with bilateral lateral lymph-node dissection was performed. Pathological evaluation confirmed the diagnosis of pT1, pN1a, M0, and pStage IIIA (UICC 8th edition), with negative resection margins (Fig. 2a). The histological and immunostaining findings were consistent with the preoperative biopsy results; immunohistochemical (IHC) was positive for human melanoma black 45 (HMB-45), S-100, Cluster of Differentiation 57 (CD57), and Melanoma Antigen (Melan A) (Fig. 2c, d).

Postoperatively, the patient underwent five courses of four-drug combination therapy at 100% of the standard dose consisting of dacarbazine, nimustine, vincristine, and interferon-beta. In addition, local administration of interferon-beta to the perianal area was performed monthly for three years, every two months during the fourth year, and every three months thereafter. Furthermore, 5-day courses of local interferon-beta administration were conducted every six months until the fifth year after surgery. Follow-up examinations included CT scans every six months and lower gastrointestinal endoscopy every two years, with the last endoscopy performed four years prior.

**Fig. 3:**
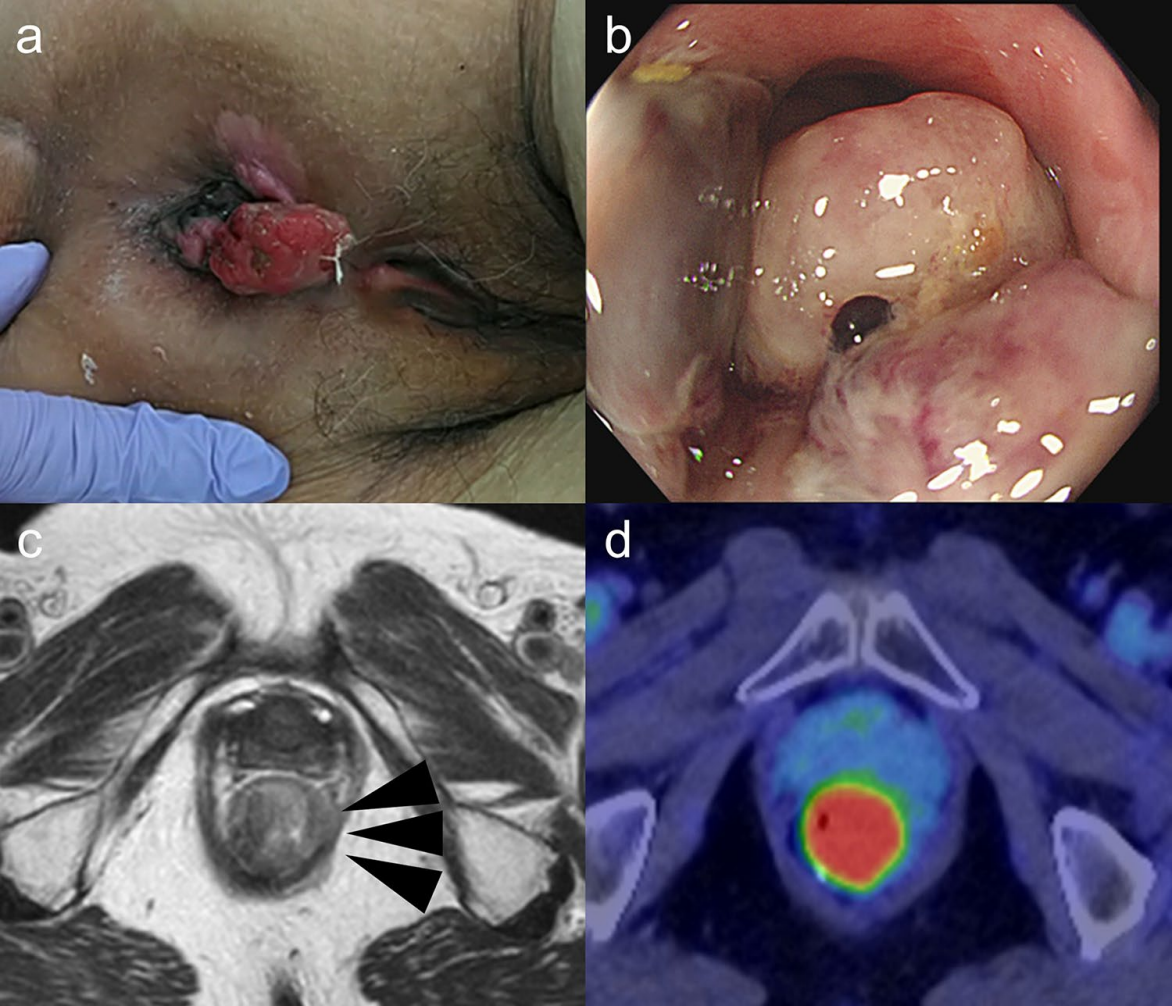
Results of the examination before the second surgical treatment. **a** Picture of the anorectal lesion. Anorectal lesion protruding from the anus. **b** Colonoscopy, black elevated lesion at the anastomotic site over the anal verge. **c** MRI findings, axial T2-weighted imaging showing a hyperintense signal. **d** PET, anastomotic lesion with an abnormal accumulation of Max SUV: 15.3

In year X, 12 years after the initial surgery, the patient returned to the clinic with a chief complaint of discomfort in the anorectal area. Physical examination revealed a hemorrhagic mass exposed to the anus (Fig. 3a). Tumor marker levels were all negative: carcinoembryonic antigen (CEA), 1.8 ng/mL; carbohydrate antigen 19–9 (CA19-9), 9 U/mL; carbohydrate antigen 125 (CA125), 9 U/mL; neuron-specific enolase (NSE), 7.59 ng/mL; and 5-S-cysteinyldopa (5-S-CD), 2.7 nmol/L.

Upper gastrointestinal endoscopy did not reveal any significant findings. Lower gastrointestinal endoscopy revealed an elevated black lesion at the anastomotic site, above the anal verge (Fig. 3b). Gastrointestinal enema identified a 2 cm elevated lesion with a gastrografin-filling defect just above the anal canal. CT revealed a highly absorbent area at the anastomotic site along with enlarged lymph nodes near the lesion. MRI revealed a circumferential lesion located in the anal canal extending from the left lateral wall of the anus to the caudal side at the level of the anastomosis site. It showed a high signal intensity on T2-weighted imaging. An enlarged lymph node was detected near the lesion (Fig. 3c). PET revealed abnormal accumulation with a maximum SUV of 15.3 at the anastomotic lesion (Fig. 3d) and multiple mildly enlarged lymph nodes in the pararectal region with an SUV of < 5.5.

The histopathological findings from endoscopic biopsy revealed atypical cells with a high nuclear-to-cytoplasm ratio, well-defined nucleoli, and melanin deposition. Immunostaining was positive for Melan A and partially positive for HMB-45, leading to a diagnosis of malignant melanoma. Based on these findings, a diagnosis of local recurrence of AMM was made, and laparoscopic APR was performed.

The surgical findings revealed no evidence of liver metastasis or peritoneal dissemination. The anastomotic site and residual rectum were laparoscopically dissected circumferentially. The levator ani muscle was dissected circumferentially using a combined laparoscopic perineal approach and the specimen was excised.

**Fig. 4:**
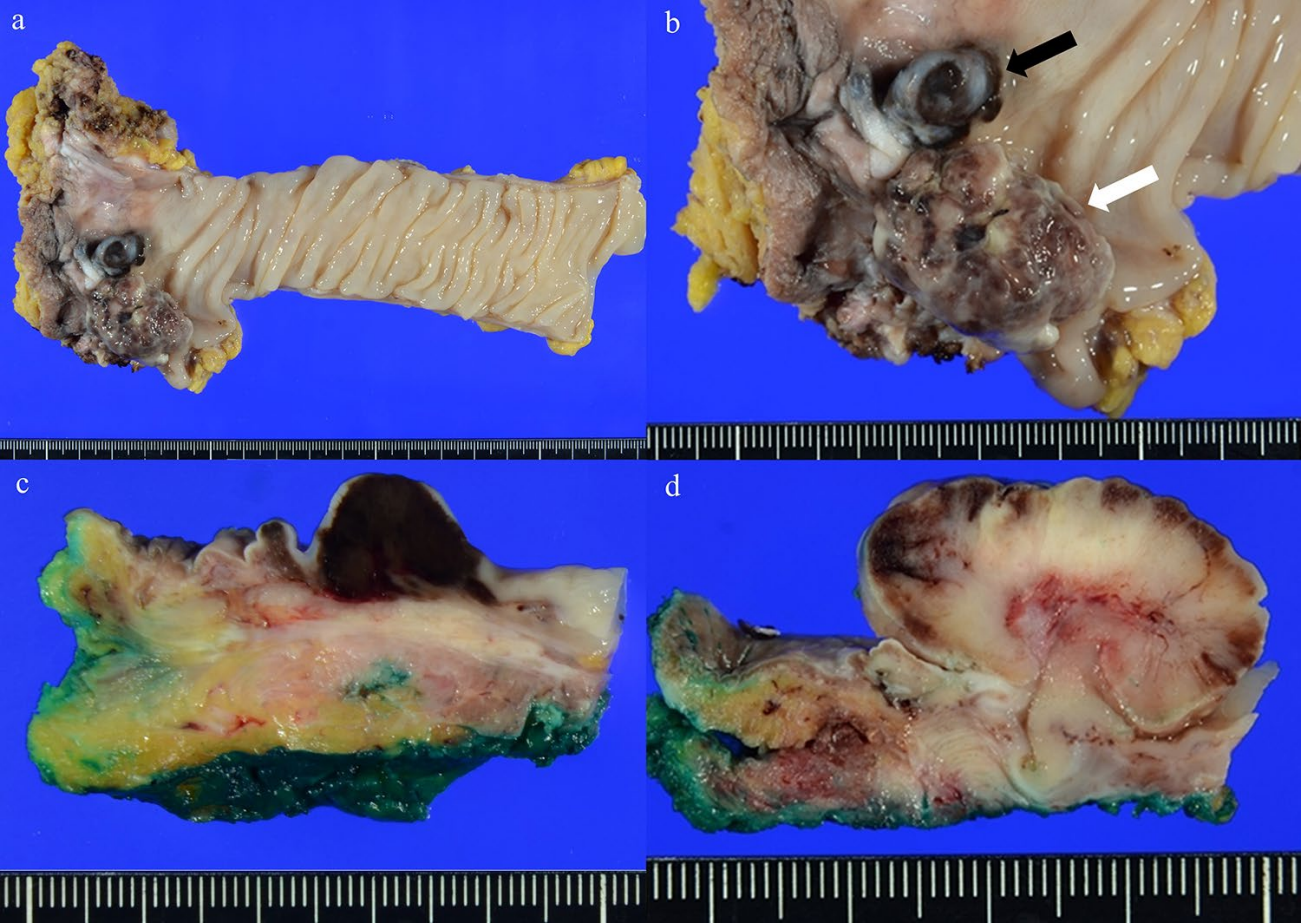
Second surgical specimen. **a**, **b** A macroscopic examination of the resected specimen revealed two elevated lesions: a melanotic lesion measuring 15 × 10 mm (black arrow) and a white lesion measuring 30 × 20 mm (white arrow). **c** The cut surface of the melanotic lesion. **d** The cut surface of the white lesion

**Fig. 5:**
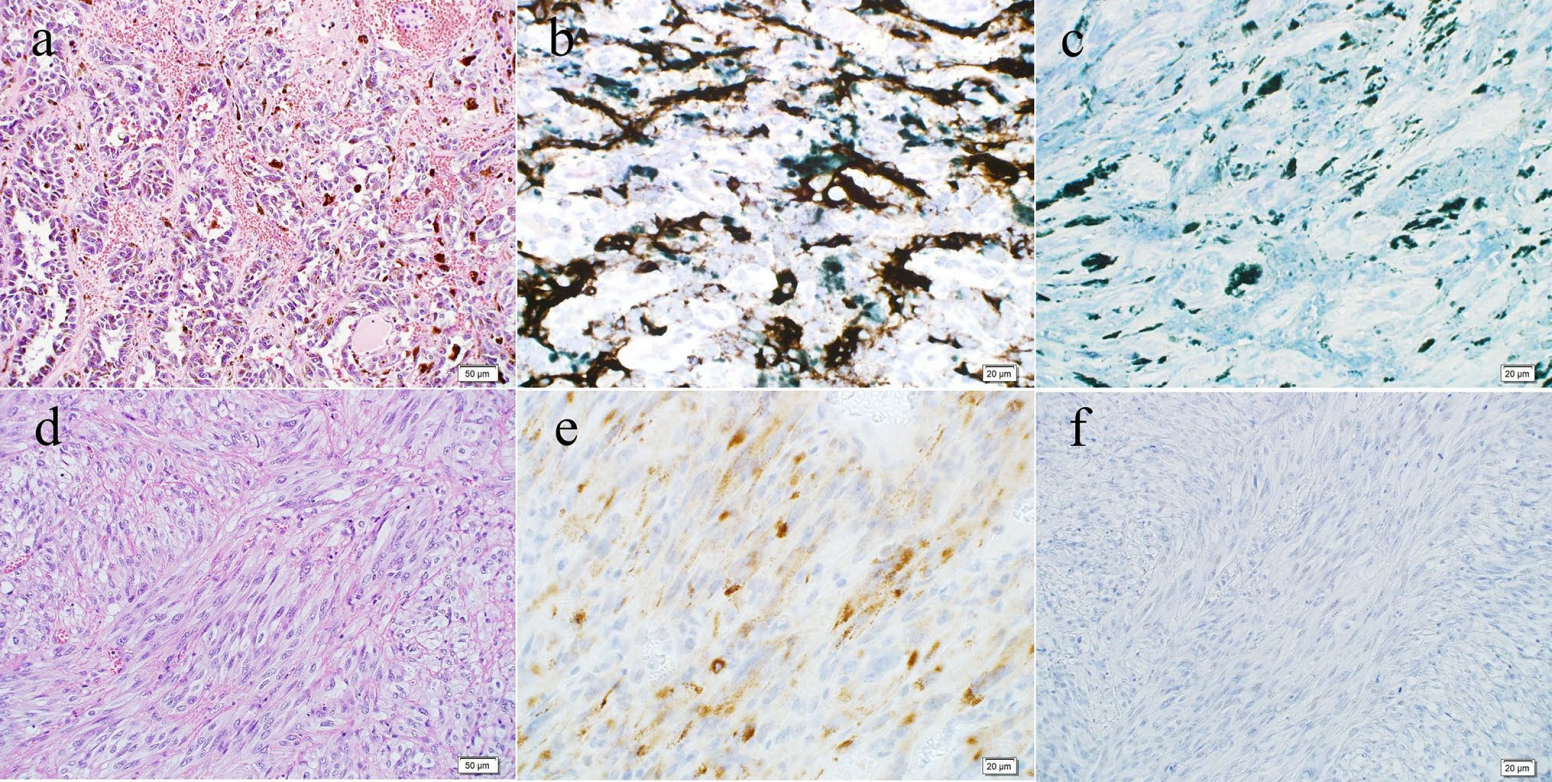
Pathohistological findings. **a** Hematoxylin and eosin staining revealed atypical cells with irregularly swollen nuclei of unequal size and distinct nucleoli with coarse chromatin form a foci-like structure and proliferate, melanin deposition was observed (magnification: 20 ×). **b** Immunohistochemical staining of melanotic lesions with Melan A was positive, whereas staining of the remaining epithelial cells was negative (magnification: 20 ×). **c** Immunohistochemical staining of melanotic lesions with HMB-45 was partially positive, whereas staining of the remaining epithelial cells was negative (magnification: 20 ×). **d** Hematoxylin and eosin staining shows white lesions containing areas composed of spindle-shaped atypical cells. Melanin deposition was unclear (magnification: 20 ×). **e** Immunohistochemical staining of the white lesion with Melan A was positive, while staining of the remaining epithelial cells was negative (magnification: 20 ×). f: Immunohistochemical staining of the white lesion with HMB-45 was partially positive, while staining of the remaining epithelial cells was negative (magnification: 20 ×)

**Fig. 6:**
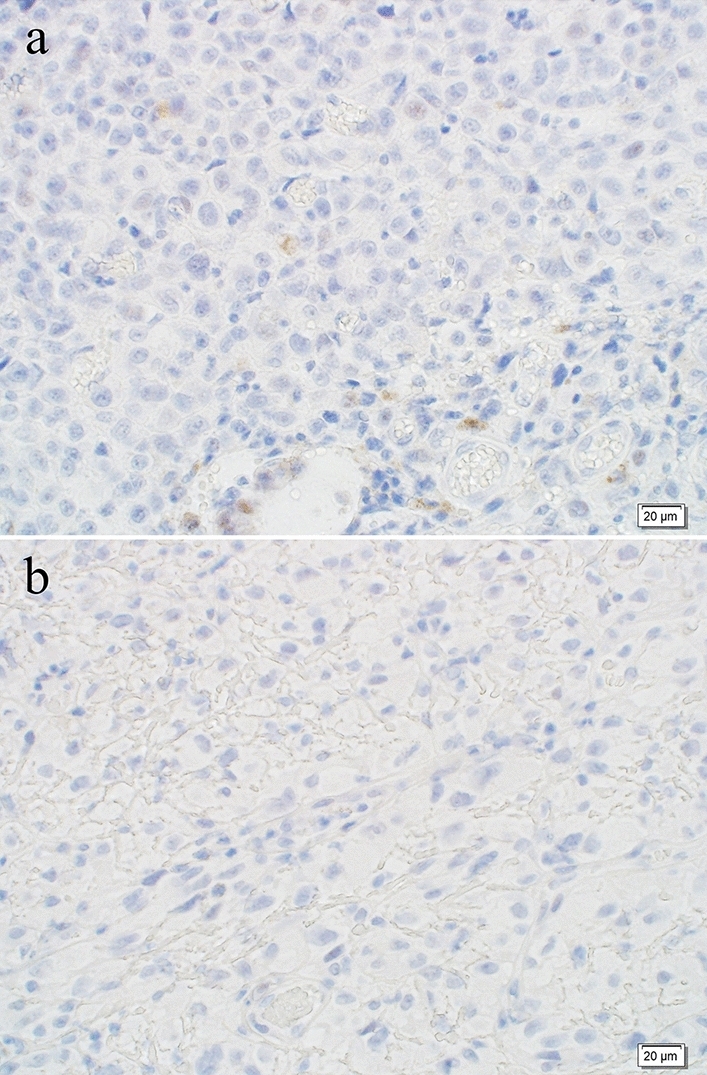
Immunostaining results of *BRAF* V600E. **a** Immunostaining of the first surgical specimen for *BRAF* V600E was negative (magnification: 40 ×). **b** Immunostaining of the surgical specimen for *BRAF* V600E was negative (magnification: 40 ×)

A macroscopic examination of the resected specimen revealed two elevated lesions: a melanotic lesion measuring 15 × 10 mm (proximal margin, 145 mm; distal margin, 25 mm; black arrow) and a non-melanotic lesion measuring 30 × 20 mm (proximal margin 120 mm, distal margin 20 mm, white arrow). In the previous surgery, anastomosis was unclear (Fig. 4). A histopathological examination revealed atypical cells with irregularly shaped nuclei of varying sizes, coarse chromatin, and distinct nucleoli in both lesions, and melanin deposition was scattered only in the melanotic lesions. (Fig. 5a). MelanA IHC was performed that showed positive in neoplastic cells (Fig. 5b). HMB-45 was partially positive in neoplastic cells (Fig. 5c). The non-melanotic lesions contained areas composed of spindle-shaped atypical cells, and melanin deposition was not evident (Fig. 5d). The IHC results were similarly positive for Melan A (Fig. 5e) and partially positive for HMB-45 (Fig. 5f). These results are consistent with those for malignant melanoma. The pathological diagnoses were pT2, pN1a, M0, and pStage IIIA (UICC for International Cancer Control 8th edition). Genetic testing was performed for the first and second surgical specimens. Both specimens were negative on anti-*BRAF* V600E antibody staining (Fig. 6). Genetic testing revealed that the specimens were negative for *BRAF* V600E/K mutations.

Postoperatively, the patient began receiving nivolumab and showed no evidence of recurrence during the 4-month follow-up period.

## Discussion

Malignant melanomas usually occur on the skin. Less than 1% of all melanomas occur in the anorectal region [1]. The age of onset is in the 60 s, and it is approximately twice as common in women as it is in men [9]. Initial symptoms include anal bleeding, anorectal mass, abnormal bowel movements, and anal pain, and the lack of specific symptoms can make diagnosis difficult [2]. This delays the initial diagnosis and therapeutic intervention, and many patients develop distant metastasis by the time a definitive diagnosis is reached. There is little evidence to support the benefits of radiotherapy or chemotherapy, and surgical resection is the first-choice treatment [2].

Many cases recur early postoperatively, with a median recurrence period of 9 months after surgical resection, with most cases recurring within 1 year [7]. The recurrence rate is as high as 65.6%, with local recurrence accounting for 20.6% of all recurrence [1, 10]. The results differed depending on the surgical technique used, with a significantly higher local recurrence rate. The rate of local recurrence after local excision in the APR group was 21.6%, while that in the local excision group was 57.7% [1].

When classified into the rectum (4–6 cm from the anal verge), anorectal transition zone (3–3.9 cm from the anal verge), and anal canal, approximately three-quarters of AMMs occur in the anal canal [11]. The high recurrence rate is thought to be partly because local resection leaves the anal canal as the preferred site of recurrence [11]. The 5-year survival rate of AMM is 19.2%, which indicates a poor prognosis [1]. The main surgical resection options are APR and local excision, but the 5-year survival rates after these surgical treatments are 18.7% and 19.9%, respectively, with no significant difference, and both are associated with a poor prognosis [1]. Therefore, cases of survival > 10 years, as in the present case, are rare, and cases of recurrence after a long period are extremely rare. A systematic literature search was conducted from inception to July 1, 2024, in PubMed, Embase, and Cochrane CENTRAL databases for AMM, and none of the 82 cases had an overall documented survival time of > 10 years [12].

In cutaneous malignant melanoma, the rate of recurrence within 10 years is 93.6%, while that after 10 years is only 6.4% [13]. In cutaneous malignant melanoma, factors, such as young age of onset, female sex, negative lymph nodes, thin tumor, absence of ulceration, and early detection at the initial diagnosis, predispose patients to recurrence after a long period [13]. In such cases, distant metastasis is more common than local recurrence, and the survival rate after recurrence is higher [13]. There are a few cases of recurrence after 10 years in AMM, and the characteristics and predictors of recurrence after a long follow-up period have not yet been fully investigated. However, since a longer survival is associated with a higher risk of recurrence after a prolonged period, favorable prognostic factors can be considered as predictors of recurrence after a prolonged period. Younger age, less-advanced disease, and fewer positive lymph nodes are good prognostic factors for overall survival [14]. In addition, women with higher transcript levels of PMCA4, an important genetic regulator of calcium, have been reported to have longer progression-free survival [15]. Further case series are needed to determine which patients are particularly susceptible to recurrence after a long disease course.

Genetically, Helmke et al. identified *BRAF* mutations in 2 of 19 (approximately 10.5%) cases of AMM (K600N and R443W) [16]. Furthermore, Taskin et al. reported a *BRAF* mutation in 1 of 15 (approximately 7%) cases of AMM (V600E) [17]. *BRAF* mutations are found in a small percentage of AMM in comparison to cutaneous melanomas. In our case, both *BRAF* V600K/E mutations were negative, as in the previous reports. In all types of melanoma, *BRAF*-mutated tumors have been reported to be more aggressive than the corresponding wild-type tumors [8]. Additionally, relapse-free survival in all malignant melanomas is longer in patients with *BRAF* V600E mutation than in those with *BRAF* V600K/V600R mutations [18]. However, no correlation with the prognosis has so far been observed for mucosal melanomas including AMM, and data restricted to AMM are scarce [19]. It is possible that a *BRAF* mutation is therefore not a poor prognostic factor, inferring from the fact that AMM is often *BRAF*-negative and it is also a disease with a poor prognosis. However, this is not clear, because there are few *BRAF* mutation-positive cases and, therefore, comparisons cannot be made. There has been insufficient research into gene mutations and the prognosis in AMM, and genetic testing needs to be performed in more cases for appropriate immunotherapy and prognostic prediction.

We experienced a rare case of anastomotic recurrence of AMM more than 10 years after the initial operation, which raises the question of whether the recurrence resulted from residual microscopic satellite lesions or represents a de novo lesion. The initial surgery was hand-stitched, and owing to the effects of the long lapse of time, the pathology did not clearly show anastomosis from the previous surgery. Therefore, it was unclear whether the lesion was located directly above or away from the anastomotic site. However, the operative records suggested that the lesion occurred directly above the anastomotic site. In addition, malignant melanoma has a wide range of histological and cytological features [20]; however, the morphology and histological findings of melanotic lesions and initial lesions are similar, while non-melanotic lesions are different. Taken together, it is possible that the melanotic lesion was a residual microscopic satellite lesion and the non-melanotic lesion was a de novo lesion. However, this could not be determined. As mentioned above, local recurrence is less common in AMM, and since distant metastasis is common in cutaneous malignant melanoma recurrence after 10 years, it is highly likely that this is a new recurrent lesion. In addition, this may have contributed to the recurrence because ISR was selected for the initial surgery, and the anal canal, a favorable site for AMM, was left in place. Long-term local follow-up is necessary when anorectal-sparing surgery is performed for malignant rectal melanomas.

## Conclusion

We encountered a case of anastomotic recurrence 12 years after intersphincteric resection of AMM and rectal amputation. Long-term local follow-up is necessary when anorectal-preserving surgery is performed for AMM.
