# Correction: A case of anastomotic recurrence 12 years after intersphincteric resection for anorectal malignant melanoma

**DOI:** 10.1007/s12328-025-02173-4

**Published:** 2025-07-05

**Authors:** Sato Nishida, Tatsuya Shonaka, Tomohiro Takeda, Masahide Otani, Mizuho Ohara, Chikayoshi Tani, Manami Hayashi, Tomoe Nakagawa, Kimiharu Hasegawa, Hideki Yokoo

**Affiliations:** 1 Division of Gastrointestinal Surgery, Department of Surgery, Asahikawa Medical University, 2-1 Midorigaoka Higashi, Asahikawa, Hokkaido 078-8510 Japan; 2 Division of Hepatobiliary Pancreatic and Transplantation Surgery, Department of Surgery, Asahikawa Medical University, Asahikawa, Japan; 3 Department of Diagnostic Pathology, Asahikawa Medical University Hospital, Asahikawa, Japan; 4 Department of Dermatology, Asahikawa Medical University, Asahikawa, Japan

**Correction: Clinical Journal of Gastroenterology** 10.1007/s12328-025-02140-z

In the caption of Fig. 2 in this article, the word ‘Fist’ should have been written as ‘First’. The original article has been corrected.

